# The Late Thomas H. Powers

**Published:** 1879-01-20

**Authors:** 


### *THE LATE THOMAS H. POWERS.*

An obituary notice of the above distinguished individual was crowded out of our last issue.

He was a leading member of the celebrated firm of Powers & Weightman, of Philadelphia. As manufacturing chemists, this firm have established a world-wide reputation.

"In looking over the characteristic traits of Mr. Powers, the one which stands out boldly, and which, without doubt, made him so successful in every enterprise, whether in business or charitable pursuits, was his wonderful penetration and foresight. His fondness and aptitude for details was remarkable. When a new matter was presented, he instinctively grasped the subject in its entirety, specifying all the elements of success, and pointing out the principal obstacles to its accomplishment. And this was true, not only as to the details of the immense business which he conducted, but applied as well to the needs of the poorest of the objects of his almost boundless charity."

The death of Mr. Powers occurred on 20th of November, at the age of 67.

